# Changes in physiological tremor associated with an epileptic seizure: a case report

**DOI:** 10.1186/1752-1947-5-449

**Published:** 2011-09-12

**Authors:** Jean-François Daneault, Benoit Carignan, Maxime Robert, Christian Duval

**Affiliations:** 1 Department of Neurology and Neurosurgery, Montreal Neurological Institute, McGill University, Quebec, Canada; 2 Département de Sciences Biologiques, Université du Québec à Montréal, Montréal, Québec, Canada; 3 Département de Kinanthropologie, Université du Québec à Montréal, Montréal, Québec, Canada

## Abstract

**Introduction:**

Epileptic seizures are associated with motor, sensory, somatosensory or autonomic symptoms that have all been described in varying detail over the years. Of interest in the present report is a case of normal physiological tremor, which to date has never been evaluated prior to and during an epileptic seizure. In fact, there is only anecdotal mention of pre-ictal and ictal changes in clinically noticeable tremor in the literature.

**Case presentation:**

Our patient was a left-handed, 27-year-old Caucasian woman diagnosed seven years previously with partial epileptic seizures, secondarily generalized. Physiological tremor was measured simultaneously on the index finger of both hands of our patient. Electromyography as well as heart rate and respiration were also monitored. A previously performed electroencephalography examination revealed abnormal oscillations focalized to the left primary somatosensory cortex. She was also diagnosed with left frontal neuronal heterotopias. We detected subclinical changes in tremor characteristics, such as amplitude, median power frequency and power dispersion, contralateral to the localization of epileptic activity. Tremor characteristics remained relatively steady ipsilateral to the localization of the epileptic activity.

**Conclusions:**

Changes in physiological tremor characteristics should be considered as another possible pre-ictal or ictal manifestation. We propose that the network associated with physiological tremor might be more sensitive to abnormal oscillations generated within the central nervous system by epileptic activity from certain structures.

## Introduction

Epilepsy is characterized by a predisposition to abnormal brain activity leading to seizures, as well as motor and non-motor symptoms [1]. In addition to motor, sensory, somatosensory or autonomic symptoms that can be encountered during the ictal phase [2], physiological changes have also been observed in the pre-ictal phase. In some cases heart rate variability has been shown to increase in the minutes prior to the clinical onset of seizures [3]. There is also anecdotal mention of pre-ictal and ictal changes in clinically noticeable tremor [4]. These manifestations are intimately linked to the localization of the seizure activity. In this report, we describe the case of an epileptic patient who had a seizure while physiological tremor (PT) was being recorded. PT is commonly described as involuntary rhythmical oscillations with sinusoidal properties. These low-amplitude oscillations, normally less than 0.5 mm [5], stem from mechanical properties of the limb [6] as well as possible central oscillators [7]. The characteristics of PT have been extensively studied by our group [8,9] and others (see [10] for review).

## Case presentation

Our patient was tested as part of a study on bilateral PT [8]. She signed the institutionally approved informed consent form, but omitted to inform us of her condition prior to testing. Detailed procedures for that study can be found in the published work [8], and are described in brief below. A medical history for our patient was gathered from her medical records. She was a left-handed, 27-year-old Caucasian woman who had been diagnosed seven years previously with partial epileptic seizures, secondarily generalized. Her medical records also described abnormal electroencephalography (EEG) oscillations focalized to the left primary somatosensory cortex. She was also diagnosed with left frontal neuronal heterotopias. Since her diagnosis, her symptoms had been effectively controlled with carbamazepine (current dose: 600 mg twice a day). Her last reported seizure occurred two years ago. Her usual pre-ictal symptoms include nausea, vomiting or headache. Ictal symptoms included numbness and involuntary contraction of the right hand and forearm that could propagate up to the neck and face. Rarely, she experienced convulsions. This indicates that the abnormal oscillations first emerged in the somatosensory cortex and propagated to motor areas as they intensify.

PT was measured simultaneously on the index finger of both hands using laser displacement sensors (LDS 90/40, LMI Technologies, Heerlen, The Netherlands). Electromyography (EMG) of the extensor digitorum communis and flexor digitorum superficialis of both forearms was recorded using bipolar, pre-amplified surface electrodes. Her heart rate and respiration were also monitored. She was seated facing a computer screen. Four conditions were planned, the first one being bilateral tremor recording while she was asked to look at a horizontally moving line on the computer screen. During that condition, the participant was asked to keep both index fingers in a horizontal position while her arms and hands were resting on a custom-designed support. Because of the seizure, the three remaining conditions were not performed. The trials lasted 60 seconds and a rest period of 60 seconds was allotted between trials. For details on analysis, see Daneault *et al. *[8].

For the first two trials, there were no reports of any physical problems or symptoms by our patient. Only after the third trial did she mention she was not feeling well. She reported slight dizziness, sweating and mentioned she was seeing spots. However, after a minute of rest she felt better and opted to continue. During the fourth trial, she identified having had a seizure. The experiment was then halted.

**Figure 1:**
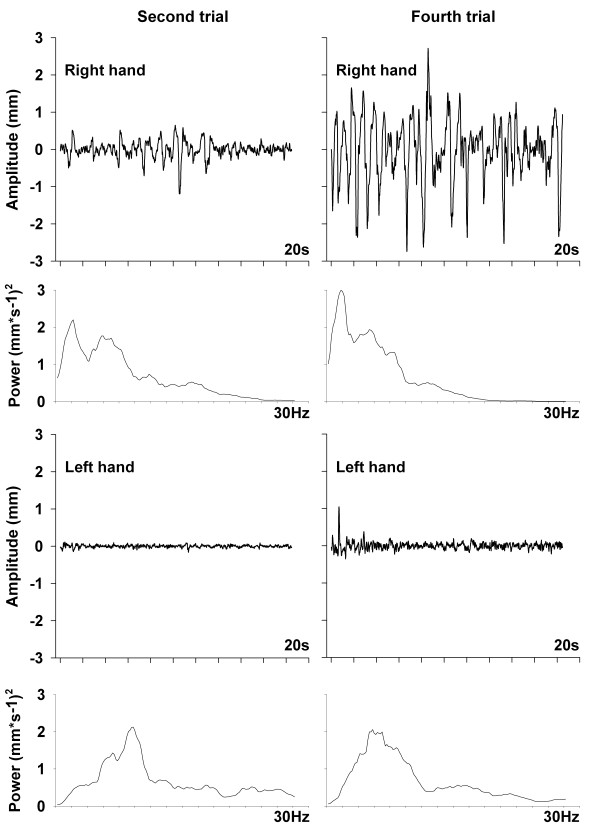
**Example of tremors and power spectra**. This graph illustrates a 20 second example of finger tremor and the resulting velocity power spectrum for both index fingers, during the second and fourth trial. Top row left: index finger tremor (20 seconds) of the right hand during the second trial. Top row right: index finger tremor (20 seconds) of the right hand during the fourth trial. Second row left: power spectrum on index finger tremor (20 seconds) of the right hand during the second trial. Note that the power spectrum was calculated on the velocity time series. Second row right: power spectrum on index finger tremor (20 seconds) of the right hand during the fourth trial. A shift of the power towards the lower frequencies in the fourth trial can be seen. Note that the power spectrum was calculated on the velocity time series. Third row left: index finger tremor (20 seconds) of the left hand during the second trial. Third row right: index finger tremor (20 seconds) of the left hand during the fourth trial. Bottom row left: power spectrum on index finger tremor (20 seconds) of the left hand during the second trial. Note that the power spectrum was calculated on the velocity time series. Bottom row right: power spectrum on index finger tremor (20 seconds) of the right hand during the fourth trial. We can observe that the spectral characteristics remain stable across the second and fourth trials. Note that the power spectrum was calculated on the velocity time series.

Analysis revealed significant changes in PT characteristics throughout the experiment. First, to illustrate the changes in PT amplitude and spectral characteristics, an example of PT of both fingers and related power spectrums in the second and fourth trials is shown (Figure 1). Note that the right hand exhibited a prominent peak at 2 Hz. However, the origin of this prominent peak, whether stemming from a change in mechanical properties or from abnormal central oscillations, cannot be addressed by the current protocol.

**Figure 2:**
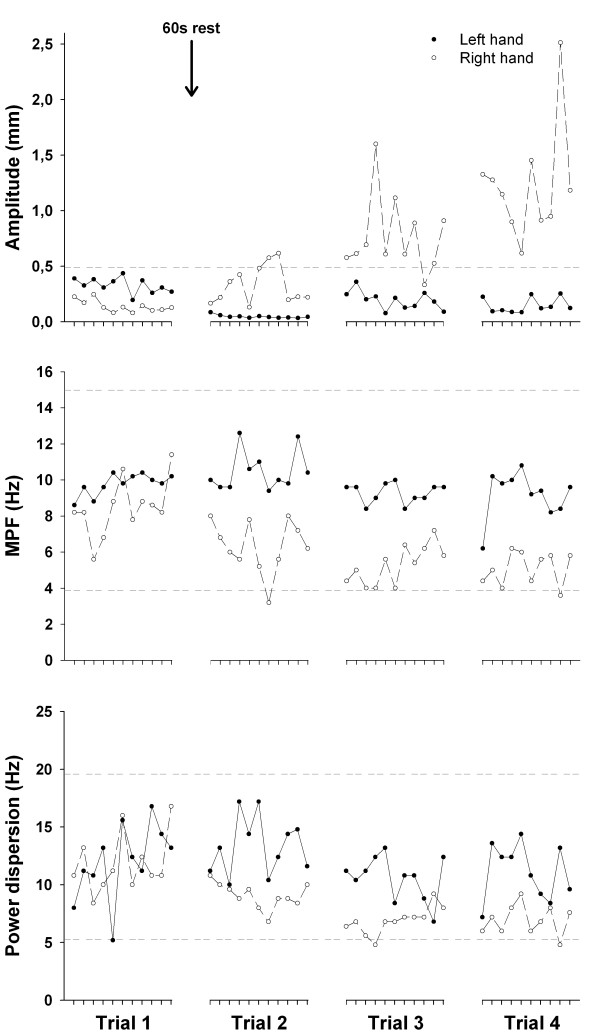
**Characteristics of physiological tremor (PT)**. This graph illustrates the amplitude, median power frequency (MPF) and power dispersion of PT in all four trials. Note that for all characteristics within each trial, all values are representative of the average of a five-second epoch. Solid dots represent values for the left index finger and white dots represent values for the right index finger. Top row: PT amplitude of both index fingers for each trial calculated on the displacement time series comprising oscillations between 1 to 30 Hz. The reference line represents the upper specification of two standard deviations of PT amplitude in 93 healthy young adults taken from a data bank [5]. This indicates that values lying below this reference line are considered within normal ranges, whereas those that lie above are considered abnormal. Middle row: MPF of both index fingers for each trial calculated on the velocity power spectrum comprising oscillations between 1 to 30 Hz. The reference lines represent the upper and lower specifications of two standard deviations of PT MPF in 93 healthy young adults taken from a data bank [5]. This indicates that values lying between those reference lines are considered within normal ranges whereas those that lie outside are considered abnormal. Bottom row: power dispersion (frequency band containing 68% of total power centered at the MPF) of both index fingers for each trial calculated on the velocity power spectrum comprising oscillations between 1 to 30 Hz. The reference lines represent the upper and lower specifications of two standard deviations of PT power dispersion in 93 healthy young adults taken from a data bank [5]. This indicates that values lying between those reference lines are considered within normal ranges whereas those that lie outside are considered abnormal. Of note is that characteristics of the left index finger remain steady throughout all four trials whereas characteristics of the right index finger begin changing as early as in the second trial.

While PT of both fingers was within normal physiological parameters during the first trial, an increase in amplitude was then observed for the right index finger (Figure 2). Although this increase was not clinically significant during the first three trials, Figure 2 clearly illustrates that the PT amplitude began to change as early as the second trial; even before our patient was conscious of any predictors such as the ones experienced after the third trial. Interestingly, PT amplitude for the left index finger remained relatively steady throughout the recordings.

Changes observed in PT amplitude were accompanied with alteration of spectral characteristics. Median power frequency (MPF) and power dispersion (frequency band containing 68% of total power centered at the MPF) both decreased for the right index finger starting in the second trial (Figure 2). MPF decreased to approach Essential tremor properties [10] while power dispersion remained within the physiological range. As for PT amplitude, spectral characteristics of the left index finger remained relatively steady throughout the recordings.

EMG results did not show any predictive signs of the imminent seizure. Although variability in EMG activity amplitude was observed between trials, no significant pattern emerged for either muscle or side. RMS values (arbitrary units) ranged from 2.27e^-4 ^to 3.04e^-4 ^for the right extensor, 4.06e^-6 ^to 1.31e^-4 ^for the right flexor, 8.63e^-5 ^to 3.81e^-4 ^for the left extensor and 2.37e^-5 ^to 5.66e^-5 ^for the left flexor.

Our patient's heart rate characteristics did not change significantly between trials; the mean RR interval was 847 ± 68.9 milliseconds, mean heart rate was 71.6 ± 6.1 beats/second, mean NN50 was 12.3 ± 4.1 spikes and mean pNN50 was 19.2 ± 5.5%. The characteristics of respiration also did not change significantly; the mean duration of every breath was 4.1 ± 0.7 seconds and the mean irregularity of the signal, that is, the standard deviation of the linear envelope of the respiration signal, was 0.7 ± 0.1. The seizure and its preceding signs did not affect the heart rate and breathing of our patient, hence they could not have been used to forecast the arrival of this epileptic seizure.

The present case illustrates that unconscious, subtle changes in PT can be observed prior to conscious signs that a seizure may occur. This is interesting since the patient's epileptiform activity originates in the somatosensory cortex and propagates to the motor areas; thus, the structures of the central network implicated in PT, whether somatosensory or motor, could be hypersensitive to abnormal oscillations. The present findings demonstrate that contralateral to the localization of the epileptic activity, while PT amplitude increased, MPF and power dispersion showed a marked decrease. These characteristics are usually associated with the presence of a dominating central oscillation (or oscillations) within the power spectrum of PT. This can be the case even while no significant change in EMG was observed because of the inherent limitations of this technique. The altered characteristics never reached extreme values that would normally be found in pathological cases such as Parkinson's or Essential tremors [9,10]. The striking feature in our patient's case is the obvious difference in PT characteristics between both hands as the seizure approached. This strongly suggests that lateralized descending pathways were involved in the changes observed and excludes systemic changes such as increased adrenaline often observed before a seizure.

PT is defined as involuntary oscillation of a limb. These oscillations stem from neural activity within the central nervous system and mechanical properties of the limb examined; all of which are modulated by reflex activity (see [10] for a review). It is believed that central oscillations are generated and propagated through a cerebello-thalamo-cortical pathway [9], whereas the mechanically-generated oscillations are a function of limb inertia and rigidity [6]. Cortical involvement in PT has long been suspected. For example, we have shown that the resurgence of central components of PT can be prevented after tremor amplitude normalization following ventrolateral thalamotomy [9]. In these cases, it was suggested that the cerebello-thalamo-cortical pathway involved in PT generation and propagation was simply interrupted. However, it is well known that the ventrobasal thalamic nucleus and not the ventrolateral thalamic nucleus synapses within the primary somatosensory cortex [11], the localization of epileptic activity in the present case. Rather, the ventrolateral thalamic nucleus synapses within motor and accessory motor areas (see [12] for a review). One simple explanation would be that altered neural activity from the somatosensory cortex was transferred to the motor regions via arcuate fibers. The findings from the present case cannot conclusively characterize the neural mechanisms involved in tremor modification, but these neural pathways are surely involved. Since PT characteristics, whether amplitude or spectral, were altered for the right side while they remained relatively steady for the left and the participant's abnormal cerebral activity was localized within her left hemisphere, we can conclude that the changes in PT characteristics were caused by this atypical brain activity. Nonetheless, we cannot exclude the possibility that central modulation of reflex activity could have caused the observed changes in PT.

The possible involvement of our patient's frontal heterotopias on the results presented here is minimal. Indeed, since this condition is chronic whereas epileptiform activity is intermittent, it would produce long-lasting alterations to PT properties. As the results clearly demonstrate, PT characteristics in the first trial were normal whereas, starting in the second and culminating in the fourth, those properties were being altered by an abnormal mechanism, namely the epileptic seizure activity.

Interestingly, only PT characteristics were significantly altered while other physiological signals, namely EMG, heart rate and respiration, remained unchanged throughout the trials. The unchanged EMG signal confirms that the recorded displacement in the fourth trial is still PT and not that our patient was swaying their right hand. As for the unchanged heart rate and respiration, this indicates a focal event, and not a generalized change in our patient's state. These results further point to the localized epileptic activity as a cause of the observed changes in PT.

Finally, we should stress that although changes in tremor characteristics have been observed with the use of anti-epileptic drugs (see [13], for example), the phenomenon observed here is quite distinct. Indeed, our patient presented with PT within normal parameters in the first trial, and although modifications of those characteristics were observed from the second to the last trial, they never entered what could be considered a pathological state. In fact, pathological tremors usually have an amplitude of more than 4 to 5 mm, with a power dispersion of less than three, such as seen in Parkinson's disease [9] or Essential tremor [10].

While the current protocol does not allow the determination of whether the changes in PT were pre-ictal or ictal, it delivers interesting information about the mechanisms of PT and perhaps into possible monitoring methods. Indeed, as PT was the only measured physiological signal modified by the epileptiform activity, which was localized to the somatosensory cortex, this can argue for a possible involvement of this cortical area in the central network generating PT. In addition, since changes in PT, although very slight, were observed prior to our patient's usual pre-ictal signs, we can hypothesize that the structures involved in the PT cortical network are hypersensitive to abnormal oscillations. Of course, studies using EEG on patients having similar types of seizures are required to confirm this hypothesis.

Also, if these observed changes were in fact pre-ictal, it could lead to the development of novel, simple and inexpensive devices able to be used for long periods of time. These could be capable of alerting individuals to an upcoming seizure before any conscious symptoms occur when an EEG is not readily available or impractical [14]. Again, further research is needed to determine the feasibility and practicality of such devices.

## Conclusions

Changes in physiological tremor characteristics should now be considered as another possible pre-ictal or ictal manifestation.

## Consent

Written informed consent was obtained from the patient for publication of this case report and any accompanying images. A copy of the written consent is available for review by the Editor-in-Chief of this journal.

## Competing interests

The authors declare that they have no competing interests.

## Authors' contributions

J-FD created the protocol, analyzed and interpreted the data from our patient regarding tremor and electromyography, and was a major contributor in writing the manuscript. BC elaborated the protocol, analyzed and interpreted the data from our patient regarding tremor and electromyography, and was a major contributor in writing the manuscript. MR interpreted the data from our patient regarding tremor and electromyography. CD interpreted the data from our patient regarding tremor and electromyography, and was a major contributor in writing the manuscript. All authors read and approved the final manuscript.
